# Dopamine Transporter (*DAT1*) and Dopamine Receptor D4 (*DRD4*) Genotypes Differentially Impact on Electrophysiological Correlates of Error Processing

**DOI:** 10.1371/journal.pone.0028396

**Published:** 2011-12-05

**Authors:** Stefanie C. Biehl, Thomas Dresler, Andreas Reif, Peter Scheuerpflug, Jürgen Deckert, Martin J. Herrmann

**Affiliations:** 1 Department of Psychiatry, Psychosomatics, and Psychotherapy, University of Würzburg, Würzburg, Germany; 2 Department of Child and Adolescent Psychiatry and Psychotherapy, University of Würzburg, Würzburg, Germany; University of Adelaide, Australia

## Abstract

Recent studies as well as theoretical models of error processing assign fundamental importance to the brain's dopaminergic system. Research about how the electrophysiological correlates of error processing—the error-related negativity (ERN) and the error positivity (Pe)—are influenced by variations of common dopaminergic genes, however, is still relatively scarce. In the present study, we therefore investigated whether polymorphisms in the *DAT1* gene and in the *DRD4* gene, respectively, lead to interindividual differences in these error processing correlates. One hundred sixty participants completed a version of the Eriksen Flanker Task while a 26-channel EEG was recorded. The task was slightly modified in order to increase error rates. During data analysis, participants were split into two groups depending on their *DAT1* and their *DRD4* genotypes, respectively. ERN and Pe amplitudes after correct responses and after errors as well as difference amplitudes between errors and correct responses were analyzed. We found a differential effect of *DAT1* genotype on the Pe difference amplitude but not on the ERN difference amplitude, while the reverse was true for *DRD4* genotype. These findings are in line with predictions from theoretical models of dopaminergic transmission in the brain. They furthermore tie results from clinical investigations of disorders impacting on the dopamine system to genetic variations known to be at-risk genotypes.

## Introduction

Electrophysiological research extensively investigates the generation of two correlates of error processing: One component, the error-related negativity (ERN), is a defined negative event-related potential (ERP) with an amplitude of up to 10 µV that appears in the response-locked EEG 50 to 100 ms after an erroneous response [1], [2]. The ERN has been theorized to reflect the activity of an error processing system that detects incorrect motor commands via a central processing pathway [3] without using sensory or proprioceptive information [2]. A second component, the error positivity (Pe), is a slow positive EEG potential that peaks 200 to 400 ms after an error is committed [1], [4]. The Pe has been suggested to represent the conscious detection of an erroneous response [3], [5], an adaptation of response strategy, or the subjective or emotional evaluation of the error [4].

An influential theoretical model by Holroyd and Coles [6] links the ERN to processes of reinforcement learning: This model holds that when an error occurs, the basal ganglia send a dopamine(DA)-mediated negative reinforcement learning signal to the anterior cingulate cortex (ACC), thereby generating the ERN.

Given the hypothesized role of DA transmission in the generation of the ERN, several studies investigated error processing in patients with Huntington's Disease (HD) and Parkinson's Disease (PD), both conditions with altered dopaminergic transmission. Beste and colleagues [7] reported reduced ERN amplitudes in HD patients with decreased amplitudes correlating with genetic disease load, thereby supporting the importance of intact striatal functioning for error processing. Results for PD patients are conflicting [8], [9], [10], which might in part be explained by an undesired side effect of dopaminergic PD medication on error processing [11].

Error processing has also been investigated in patients with psychiatric disorders, with most research focusing on attention deficit hyperactivity disorder (ADHD), a disorder which comprises altered striatal dopamine levels [12]. Many studies report reduced Pe amplitudes in ADHD patients, a reduction of ERN amplitudes, however, does not seem consistent across studies [13], [14], [15], [16].

In healthy volunteers, pharmaceutical alteration of dopaminergic transmission in the basal ganglia by a single dose of d-amphetamine resulted in enlarged ERN amplitudes, which was attributed to d-amphetamine's agonistic effect on dopaminergic transmission [17]. In turn, reducing dopaminergic transmission in the basal ganglia by using the antipsychotic D2/D3 receptor antagonist haloperidol led to a significant reduction of ERN amplitude in healthy participants [11], [18], [19].

So far, only few studies of common genotype variations that influence dopaminergic transmission have been reported and these are partly contradictory (for a review see [20]). The catechol-*O*-methyltransferase (*COMT*) polymorphism, for example, is known to influence frontal dopamine levels [21] and was found to affect Pe but not ERN amplitude in one study [22], whereas another study [23] did not yield significant findings. The second study, however, found an effect of a single nucleotide polymorphism (SNP) that supposedly leads to a reduction of dopamine D4 receptors (DRD4) on ERN but not Pe amplitude [23]. In addition, functional magnetic resonance imaging studies investigated genetic polymorphisms influencing dopamine D2 receptor (DRD2) expression: These receptors are prominently expressed in the basal ganglia and genetic variations seem to influence the ability to learn from negative feedback [24], [25].

Since previous research seems to support the hypothesized crucial role of dopaminergic signalling in error processing, we decided to investigate two new polymorphisms of important genes that influence dopaminergic transmission in different areas of the brain:

The gene encoding the dopamine transporter (*DAT1, SLC6A3*) was found to possess a genetic variation – a 40 nucleotide variable number of tandem repeats (VNTR) polymorphism in the 3′ untranslated region (UTR) – with two common alleles labelled *9-repeat* (*9R*) and *10-repeat* (*10R*), which supposedly influence gene expression and thereby DA reuptake from extracellular space [26], [27], [28], [29], [30]. The direction of this influence, however, is not yet clear, with studies yielding differing results with regard to which VNTR results in greater DAT expression. The DAT is primarily expressed in the striatum with only scarce expression in the prefrontal and medial frontal cortex [31], [32]. Two in vivo SPECT studies of healthy adults showed that the presence of at least one *9R* allele led to increased DAT availability in the participants' striatum [33], [34], which might indicate increased striatal dopamine availability in *10/10R* carriers.

The dopamine D4 receptor gene (*DRD4*) also possesses a polymorphism with a 48 bp variable number of tandem repeats (VNTR) in exon III, which can vary from 2 to 11 repeats [35], [36]. An investigation of human post-mortem brain tissue recently showed a trend for reduced DRD4 mRNA expression in samples carrying at least one seven-repeat allele [37]. DRD4 is mainly expressed in the prefrontal cortex with low levels of expression in the basal ganglia [36], [38].

Based on the model by Holroyd and Coles [6] as well as previous research outlined above, it is reasonable to expect some influence of these genes on error processing components: The *DAT1 10/10R* genotype was linked to smaller Pe amplitudes in children with ADHD, but showed no effect on the ERN [39]; a study of the *DRD4 SNP -521C/T* found increased ERN amplitudes for homozygous carriers of the *T* allele, which supposedly leads to less transcriptional efficiency [23].

In our experiment, we examined ERN and Pe amplitudes during a simple reaction time task in an unselected sample of healthy participants and compared amplitudes according to participants' *DAT1* and *DRD4* genotypes. Based on previous research, we expected the *DAT1 10R* genotype to be linked to smaller Pe and the *DRD4 7R* genotype to be linked to increased ERN amplitudes.

## Methods

### Ethics Statement

Ethical approval was obtained through the Ethical Review Board of the medical faculty of the University of Würzburg (vote 131/04); all procedures involved were in accordance with the 2008 Declaration of Helsinki. Participants gave written informed consent after full explanation of the procedures.

### Participants

One hundred sixty subjects (70 male, 90 female) participated in this study. Mean (*M*) age was 27.02 years, ranging from 20 to 50 years of age (standard deviation *SD*  = 7.28). All participants were right-handed, without any medication, and never treated for neurologic or psychiatric problems. Results from a subsample (n = 56) have been published elsewhere [16], [40].

Participants were stratified into a homozygous *10/10R* group (n = 98) and a group carrying at least one *9R* allele (9/*9R* or *9/10R*; n = 62) according to their *DAT1* VNTR genotype. Additionally, participants were stratified based to their *DRD4* VNTR genotype into a *7R* group (at least one *7R* allele; n = 46) and a no *7R* group (n = 114). *DAT1* and *DRD4* subgroups did not differ significantly with respect to gender, age, symptoms of depression, reaction time for correct answers, number of errors, and number of trials without artefacts per condition (as assessed with t-tests, all *p*>.05). The severity of ADHD symptoms as assessed with the ADHD Self-Report Scale (ASRS) differed between *DAT1* subgroups, with increased values for the *9R* group compared to the *10/10R* group (*t*_(158)_ = 2.66, *p* = .009; see table 1 for details).

**Table 1:** Mean and standard deviation of demographic data, psychiatric symptoms, reaction time in milliseconds, and artefact-free trials.

	*DAT1*		*DRD4*	
	*9R*	*10/10R*	*no 7R*	*7R*
n (female)	62 (31)	98 (59)	114 (66)	46 (24)
Age	27.34 (*7.56*)	26.82 (*7.14*)	27.11 (*7.64*)	26.80 (*6.40*)
Symptoms of depression	3.29 (*3.41*)	4.36 (*3.93*)	3.93 (*3.92*)	3.98 (*3.36*)
ADHD symptoms	**24.93 (** ***8.22*** )*	**21.62 (** ***7.17*** )*	22.75 (*7.54*)	23.23 (*8.30*)
Reaction time	448.57 (*62.81*)	460.50 (*52.38*)	455.21 (*60.63*)	457.54 (*46.35*)
Number of errors	59.48 (*38.12*)	52.53 (*30.78*)	55.15 (*34.66*)	55.41 (*32.18*)
Artefact-free trials correct	261.21 (*66.25*)	260.54 (*41.50*)	260.89 (*53.78*)	260.57 (*48.98*)
Artefact-free trials incorrect	55.42 (*36.26*)	49.94 (*30.79*)	51.41 *(33.80*)	53.67 (*31.26*)

Standard deviations are shown in parentheses; significant differences are marked with *.

### Psychological Assessment

Participants completed an 18 item screening questionnaire [41] based on the diagnostic criteria of ADHD as stated in the DSM-IV-TR [42]. It measures the frequency of ADHD symptoms ranging from 0 (‘never’) to 4 (‘very often’). Additionally, the Beck Depression Inventory (BDI, [43]) was used to assess depressive symptoms.

### Stimuli and Procedure

We used a modified version of the Eriksen Flanker Task [44] as published before [16], [40]. Since the focus of this study was on neural correlates of error processing, the task was adapted to obtain high error rates. One of four possible combinations of arrows (<<><<, >><>>, ><><>, <><><) was shown in the middle of a 15′ monitor for 125ms. Stimuli were presented in random order, with the probability of occurrence being .25 for each combination. Subjects completed 160 trials twice, with a short break in between, where they had to indicate the direction of the middle arrow as quickly and as accurately as possible with their left (<) and right (>) index finger, respectively. Feedback was given 750 ms after the response by showing a plus sign (correct response), a minus sign (incorrect/no response), or an exclamation mark (correct response outside the reaction time limit) in the centre of the screen for 500 ms. The interstimulus interval varied between 500 ms and 1000 ms. The reaction time limit was determined individually in a practice session – which consisted of 40 trials with a 500 ms time limit – and the mean reaction time over all correct trials was adapted as reaction time limit for the subsequent experimental task. This time limit served to increase error rates. However, all responses (including “late” responses) were subsequently analyzed.

### EEG Acquisition and Analyses

Event-related potentials were recorded from 26 Ag/AgCl scalp electrodes. Besides the recording sites specified in the 10–20 system, electrodes were placed on Oz, Fpz, under the right eye and on its outer canthus, and on the left and right mastoids. Impedance was kept below 5 kΩ for all electrodes. The ground electrode was placed between Fz and Fpz. Sampling rate was 1000 Hz, with the amplifier band pass filter set to 0.1–70 Hz and the notch filter set to 50 Hz. All data were recorded in relation to a midline reference placed between Cz and Fz and re-referenced offline to an average reference.

Eye movement artefacts were corrected [45] and response-locked EEG epochs from −200 ms to 500 ms were defined separately for correct and incorrect responses. Artefact-free epochs (no voltage in any channel exceeding ±100 µV or showing drops or rises of more than 100 µV/ms) were averaged: For incorrect responses, an average of 52.06 (*SD*  = 33.01) epochs and for correct responses, an average of 260.80 (*SD*  = 52.29) epochs were averaged.

Baseline correction based on the mean amplitude from −200 ms to 0 ms was implemented, and the time windows to detect ERN and Pe peaks were subsequently determined based on the grand average time curve over all subjects. The ERN was then automatically identified as the negative peak value between −35 ms and 108 ms over the electrode position Cz; the Pe was analogically detected as the positive peak value between 110 ms and 450 ms.

ERP amplitudes were analysed separately for the ERN and the Pe by using an analysis of variance (ANOVA) with the factors condition (correct, incorrect) and genotype (*DAT1* (*9R* group vs. *10/10R* group) and *DRD4* (no *7R* vs. *7R*)). Two-sided t-tests were used for post-hoc analyses. For all analyses, *p*-values below .05 were considered significant.

### Genotyping

Genomic DNA was extracted from whole blood samples by salt precipitation according to standard protocols. Genotyping of the *DAT1* VNTR was performed by polymerase chain reaction (PCR) and subsequent gel electrophoresis as published previously [46]. Genotyping for the *DRD4* VNTR was accomplished using standard PCR procedures modified from a previously published protocol [47]. Further details on protocols are available upon request.

## Results

There was a main effect for response time following an erroneous as compared to a correct response (*F*_[1,156]_  = 90.25, *p*<.001; post-correct responses: *M* = 452.21 ms, *SD* = 56.41; post-error responses: *M* = 476.90 ms, *SD* = 68.01), showing significant post-error slowing across all genotype groups. There was no main effect of *DAT1* genotype (*F*_[1,156]_ = .82, *p* = .366) or *DRD4* genotype (*F*_[1,156]_ = .05, *p* = .831) and no interaction effects.

Participants gave an average of 267.87 correct responses (*SD* = 51.13). There was no main effect of *DAT1* genotype (*F*_[1,156]_ = .16, *p* = .690) or *DRD4* genotype (*F*_[1,156]_ = 1.01, *p* = .318) as well as no interaction effects.

For the ERN, we found a main effect of condition (*F*_[1,156]_ = 144.96, *p*<.001) with incorrect responses leading to more negative values than correct ones and an interaction effect of condition and *DRD4* genotype (*F*_[1,156]_ = 5.37, *p* = .022), which is discussed below. We did not find an interaction effect of condition and *DAT1* genotype (*F*_[1,156]_ = .40, *p* = .526), or a three-way interaction effect of condition, *DAT1* genotype, and *DRD4* genotype (*F*_[1,156]_ = .66, *p* = .419). In addition, no main effects of *DAT1* genotype (*F*_[1,156]_ = .73, *p* = .394) or *DRD4* genotype (*F*_[1,156]_ = .17, *p* = .685) were found.

**Figure 1:**
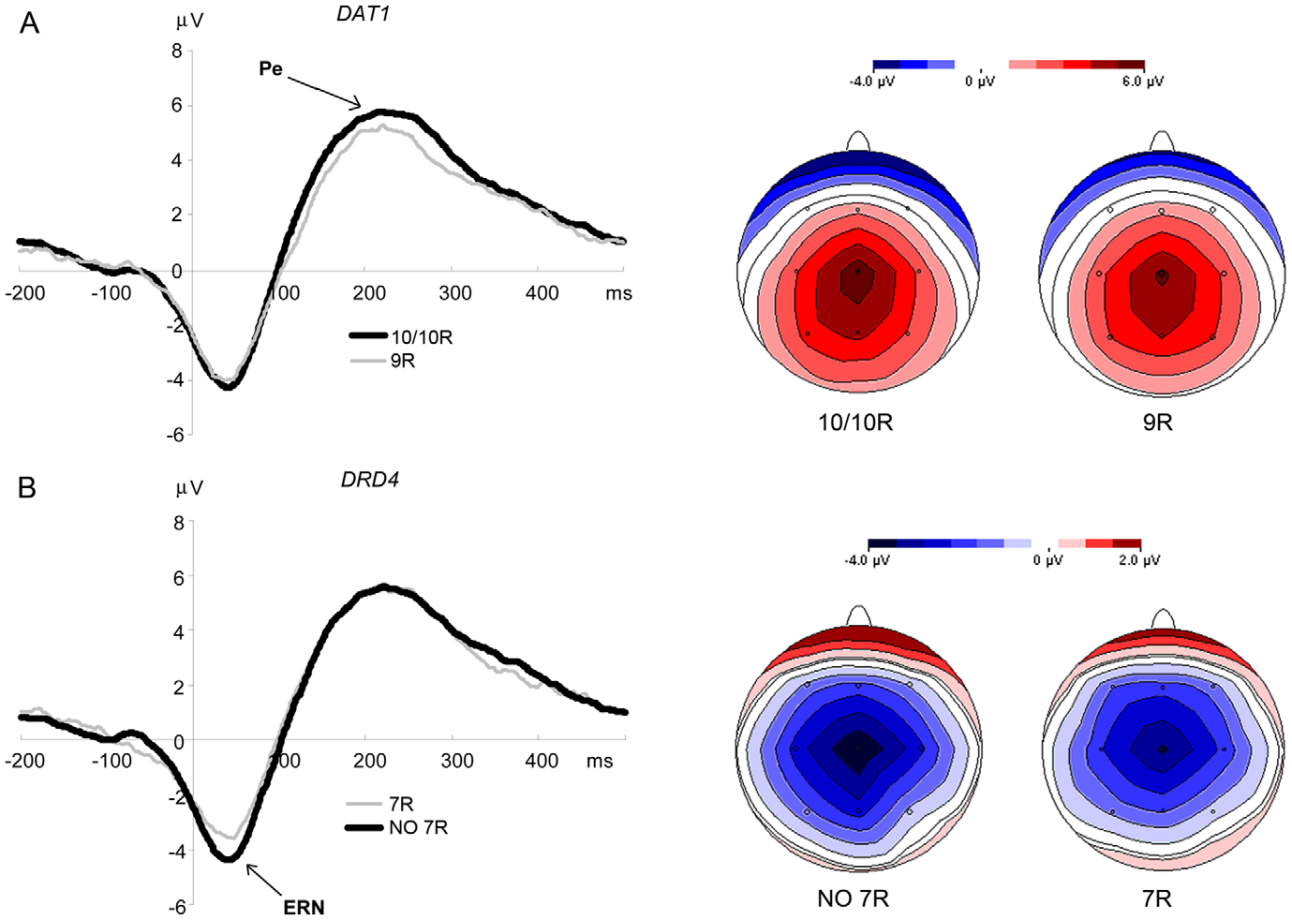
Grand average difference curves and peak topographies for *DAT1* (A) and *DRD4* (B) subgroups. Peak topographies for the ERN are at 40 ms, for the Pe at 218 ms.

The significant interaction effect of condition and *DRD4* genotype can be described by significantly smaller difference amplitudes between the correct and incorrect response conditions in *7R* carriers compared to no *7R* carriers (*t*_(158)_ = −2.18, *p* = .031; see table 2 for *M*, *SD*, and post-hoc t-tests; figure 1).

**Table 2:** Mean and standard deviation of the amplitudes for the different conditions and genotypes.

	*DAT1*			*DRD4*		
	*9R*	*10/10R*	*t* ^1^-value (*p*)	no *7R*	*7R*	*t* ^1^-value (*p*)
ERN						
– correct	1.01 (*2.22*)	.70 (*2.56*)	.77 (.442)	.95 (*2.34*)	.49 (*2.66*)	1.08 (.281)
– incorrect	−2.78 (*3.06*)	−3.18 (*3.43*)	.76 (.451)	−3.24 (*3.29*)	−2.48 (3.27)	−1.32 (.187)
– difference	−3.78 (*3.30*)	−3.88 (*3.23*)	.189 (.850)	−4.19 (*3.01*)	−2.98 (*3.65*)	−2.18 (.031)*
Pe						
– correct	5.16 (*3.55*)	4.12 (*3.68*)	**1.77 (.079)^+^**	4.53 (*3.41*)	4.52 (*4.25*)	.02 (.984)
– incorrect	8.96 (*3.24*)	8.99 (*3.64*)	−.05 (.957)	8.92 (*3.45*)	9.12 (*3.59*)	−.32 (.753)
– difference	3.80 (*3.37*)	4.87 (*3.36*)	−**1.97 (.051)^ +^**	4.40 (*3.24*)	4.60 (*3.78*)	−.34 (.731)

Standard deviations are shown in parentheses; significant differences are marked with *, trends are marked with ^+^; ^1^ df = 158, two-sided tests.

For the Pe, we also found a main effect of condition (*F*_[1,156]_ = 197.70, *p*<.001) with incorrect responses leading to more positive values than correct ones and an interaction effect of condition and *DAT1* genotype (*F*_[1,156]_ = 4.78, *p* = .030), which is discussed below. We did not find an interaction effect of condition and *DRD4* genotype (*F*_[1,156]_ = .01, *p* = .972) or a three-way interaction effect of condition, *DAT1* genotype, and *DRD4* genotype (*F*_[1,156]_ = .99, *p* = .321). Furthermore, no main effects of *DAT1* genotype (*F*_[1,156]_  = .34, *p* = .562) or *DRD4* genotype (*F*_[1,156]_ <.001, *p* = .987) were found.

The significant interaction effect can be described by marginally smaller difference amplitudes between the correct and incorrect response conditions in *9R* carriers (*t*_(158)_ = −1.97, *p* = .051; see table 2). A tendency for increased amplitudes of the Pe to correct reactions in *9R* carriers (*t*_(158)_ = 1.77, *p* = .079; see table 2 and figure 1) suggests that the interaction effect was mainly but not solely caused by differences in the processing of correct responses.

## Discussion

We found the ERN amplitude to be related to participants' *DRD4* genotype but not to their *DAT1* genotype, whereas the reverse was true for the Pe amplitude. It therefore seems that the *DRD4* genotype influences the ERN – defined as the difference wave between incorrect and correct responses – but not the Pe. In turn, *DAT1* genotype seems to influence the Pe – also defined as the difference wave – but not the ERN.

According to the model by Holroyd and Coles [6] mentioned above, the amplitude of the ERN should be influenced by phasic changes in dopaminergic transmission in the basal ganglia. Although our results seem to contradict this prediction – DRD4 is mostly expressed in the cortex with very little expression in striatal areas [36], [38] – Rubinstein and colleagues [48] discovered that mice lacking dopamine D4 receptors showed elevated dopamine synthesis and conversion in the striatum. This was possibly caused by glutamatergic transmission from cortical regions and still has to be investigated in humans. It would, however, offer a plausible explanation for our findings.

Based on Bilder and colleagues' [49] model of tonic and phasic dopamine transmission, lower cortical expression of the DRD4 in *7R* carriers could result in compensatorily elevated tonic dopamine levels in the striatum. This elevation in tonic striatal dopamine would make *7R* carriers less sensitive to changes in phasic dopamine transmission, which could explain their reduced ERN amplitude. A similar argument was proposed by Krämer and colleagues [23], who found an increased ERN amplitude to be related to the *DRD4 SNP -521C/T*. However, while the impact of this SNP on gene expression is still under debate [50], [51] we can be fairly confident about the transcriptional consequences of the VNTR we investigated.

The results can also be supported from a clinical perspective: ERN amplitude has been connected to self-reported impulsivity with highly impulsive individuals showing lower amplitudes [52]. Impulsivity, on the other hand, was recently linked to the presence of at least one *7R* allele [53]. It would therefore seem logical to find lower ERN amplitudes in *7R* carriers.

Regarding *DAT1*, our findings seem to be at odds with a study in children that found smaller Pe amplitudes in *10/10R* carriers [39]. The *10R* allele has been associated with childhood ADHD [54] which in turn has been associated with decreased Pe amplitudes [13], [14], [55]. Recent studies, however, point to a differential impact of this VNTR in childhood as compared to adulthood [56], [57]: In adults, carrying the *9R* allele was associated with persistent ADHD and worse cognitive functioning, respectively.

In addition, a recent SPECT study reported a *9R* haplotype which showed significantly higher DAT expression than all other investigated haplotypes [34]. This led the authors to suggest that reported higher striatal dopamine transporter availability in *9R* carriers could mainly be caused by this specific subgroup. It might therefore be interesting and more informative to investigate Pe amplitudes in participants carrying this haplotype and compare them to all other haplotypes.

The findings of van de Giessen and colleagues [34] underscore the importance of basic research into the effects of VNTRs and SNPs as well as haplotypes on gene expression. While the interpretation of our results relies on the findings of these studies, reports on the effects of a certain genetic variation on gene expression are still often contradictory.

Limitations of the present study are linked to the sample size: Although representing a rather large sample in neurophysiological genetic research, power considerations arise from a general genetic research perspective. The sample size seemed sufficient assuming an intermediate effect size, and effect sizes of Cohen's *d* = .38 were found for the ERN amplitude depending on participants' *DRD4* genotype and of *d* = .32 for the Pe amplitude depending on participants' *DAT1* genotype. A post-hoc power calculation (two-tailed) revealed a probability of detecting a true significant difference of 58 and 50 percent, respectively. For the non-significant findings, effect sizes were *d*≤.06, indicating that these results represent non-meaningful and practically irrelevant effects.

Furthermore, it would have been interesting to investigate gene-gene interactions. However, this procedure would have resulted in four – partly very small – subgroups, which would likely obscure the effects. Even though this could therefore not be implemented, both our *DAT1* and *DRD4* groups are very comparable with regard to demographic data, psychiatric symptoms, reaction time, and artefact-free trials. The only group difference was found for the *DAT1 9R* and *10/10R* groups with regard to their symptoms of ADHD. This is in line with the current literature [56], [57], and further helps to tie clinical findings of reduced Pe amplitudes in ADHD to an identified genetic risk factor.

Although we did not find any results on the behavioral level, our study is able to link previously observed error processing deficits in impulsive and ADHD participants to genetic variants that are known to contribute to these disorders.
